# Navigating the Abyss: Psychological Burdens in Brain Tumor Patients

**DOI:** 10.5281/zenodo.15845381

**Published:** 2025-07-11

**Authors:** Ming-Chun Yu, Orwa Aboud

**Affiliations:** 1 Department of Neurology, Creighton University School of Medicine, Omaha, USA; 2 Department of Neurology, University of California Davis, Sacramento, USA; 3 Comprehensive Cancer Center, University of California, Davis, Sacramento, USA; 4 Department of Neurosurgery, UC Davis Comprehensive Cancer Center, University of California Davis, Sacramento, USA

## Abstract

The psychological burden of brain tumors extends far beyond the physical symptoms. This article sheds light on the deep emotional and mental challenges experienced by a patient with Glioblastoma, underscoring the urgent need for comprehensive psychological support as a vital component of neuro-oncology care.

## INTRODUCTION

Brain tumors are often viewed primarily through the lens of physical symptoms-seizures, motor deficits, cognitive decline, and more. However, the psychological toll these diagnoses inflict can be equally, if not more, devastating [1]. In the journey from diagnosis to treatment, patients grapple not only with the impending loss of physical function but also a profound struggle with the erosion of their identity, autonomy, and future aspirations [2]. This article recounts the story of a distinguished dentist whose battle against Glioblastoma illustrates the deep emotional challenges encountered by brain tumor patients.

## CASE PRESENTATION

Dr. A, a 79-year-old right-handed dentist, was a pillar in his community, admired for his precision and care in his profession. He led a life marked by accomplishment and independence —until one day, he noticed a subtle weakness in his left hand, initially dismissing it as fatigue. The weakness soon spread and was accompanied by a facial droop. Concerned, he sought medical attention, leading to the discovery of a right frontal mass which was later confirmed as Glioblastoma, WHO grade 4.

The mass in the motor cortex posed a direct threat to Dr. A’s hand function that is essential to his career. The diagnosis marked the beginning of a grueling journey of surgery, radiation, and chemotherapy, while the psychological burden was equally taxing.

### Psychological impact

As the physical symptoms progressed, the emotional burden intensified. Dr. A, once a man of capability and self-reliance, found himself increasingly dependent on others for even basic tasks. The loss of autonomy was devastating. The man who had once climbed onto his roof to clear gutters now struggled to lift a fork. The growing sense of loss and helplessness culminated in moments of profound despair.

In a particularly dark period, Dr. A considered taking his own life. His plan was meticulous and tragic—he would fly his private jet westward over the Pacific Ocean into the setting sun until the fuel ran out, and the plane would plunge into the sea. To him, this represented a controlled and fitting end to a life once characterized by precision and autonomy. Yet, the legal and financial implications of such an act gave him pause. Without a body, the issuance of a death certificate could take up to 6 years, leaving his family in financial limbo. This realization deterred him from following through, but the psychological wounds remained profound.

## DISCUSSION

Dr. A’s story is far from unique among brain tumor patients. A tumor in the central nervous system is often found after the onset of neurological symptoms, and neurological deficit may persist or progress. Impairing physical function, autonomy and self-reliance lead to loss of identity, as patients can no longer engage in the activities that once defined them. A brain tumor diagnosis poses a profound existential threat, disrupting patients’ sense of self, their future, and their relationships [3]. This loss is compounded by the knowledge that their condition is likely to worsen, leading to increased dependency and suffering [4].

The psychological stressors faced by brain tumor patients are manifold—fear of death, loss of independence, altered body image, and concerns about burdening loved ones [5,6]. These stressors can lead to significant anxiety, depression, and even suicidal thoughts, as seen in Dr. A’s case. Despite multi-disciplinary management is recommended in current oncologic practice and has been shown to improve outcomes of several cancers in neuro-oncology [7], such psychological aspects are often under-recognized and inadequately addressed in clinical care.

## CONCLUSION

Dr. A’s story highlights the need for incorporating psychological support into neuro-oncology care, including routine mental health assessments. Counseling, support groups, and psychiatric care should be integral, addressing the emotional challenges that come with the diagnosis.
